# Morphine promotes the malignant biological behavior of non-small cell lung cancer cells through the MOR/Src/mTOR pathway

**DOI:** 10.1186/s12935-021-02334-8

**Published:** 2021-11-25

**Authors:** Xingyun Liu, Jia Yang, Chengwei Yang, Xiang Huang, Mingming Han, Fang Kang, Juan Li

**Affiliations:** 1 Department of Anesthesiology, Anhui Provincial Hospital Affiliated to Anhui Medical University, Hefei, 230036 China; 2 Department of Anesthesiology, The First Affiliated Hospital of USTC, Division of Life Sciences and Medicine, University of Science and Technology of China, Hefei, 230036 China

**Keywords:** Morphine, Non-small cell lung cancer, µ-Opioid receptor, Malignant biological behavior, Src/mTOR

## Abstract

**Background:**

Morphine, a µ-opioid receptor (MOR) agonist, has been shown to be related to the activity of cancer cells, and a higher morphine dosage reduces the survival time of patients with lung cancer. However, the effect of morphine on the malignant behavior of lung cancer cells remains unclear. The aim of this study was to investigate the specific molecular mechanism by which morphine regulates the malignant biological behavior of non-small cell lung cancer.

**Methods:**

Immunofluorescence staining and Western blot analyses were performed to detect MOR expression. H460 non-small cell lung cancer cells were used in this study, and cell proliferation, the cell cycle and apoptosis were evaluated using Cell Counting Kit-8 (CCK-8) and flow cytometry assays, respectively. Cell migration and invasion were detected using wound healing and Transwell assays. The effect of morphine on lung cancer development in vivo was examined by performing a xenograft tumor assay following morphine treatment.

**Results:**

Morphine promoted the growth of H460 cells both in vivo and in vitro. Morphine enhanced cell migration and invasion, modified cell cycle progression through the S/G_2_ transition and exerted an antiapoptotic effect on H460 cells. Additionally, morphine increased Rous sarcoma oncogene cellular homolog (Src) phosphorylation and activated the phosphoinositide 3 kinase (PI3K)/protein kinase B (AKT)/mammalian target of rapamycin (mTOR) pathway. Treatment with the MOR antagonist methylnaltrexone (MNTX) and the Src inhibitor protein phosphatase 1 (PP1) reduced the phosphorylation induced by morphine. Furthermore, MNTX, PP1, and the PI3K/AKT inhibitor deguelin reversed the antiapoptotic effect of morphine on lung cancer cells.

**Conclusion:**

Morphine promotes the malignant biological behavior of H460 cells by activating the MOR and Src/mTOR signaling pathways.

**Supplementary Information:**

The online version contains supplementary material available at 10.1186/s12935-021-02334-8.

## Introduction

Lung cancer is the second most common cancer worldwide and the leading cause of cancer-related mortality. More than 2 million new cases of lung cancer were reported worldwide in 2020, with at least 1.7 million patients dying from lung cancer [1, 2]. Non-small cell lung cancer (NSCLC) accounts for 85–90% of all cases, and the main treatment methods include surgery, radiation therapy, chemotherapy, and immunotherapy [3]. Radical resection is the only possible treatment for patients with lung cancer, but the prognosis of patients with advanced lung cancer is still poor. Morphine is a commonly used clinical drug for both surgical resection and pain treatment in patients with advanced lung cancer [4]. It exerts direct effects on the viability and migration of breast cancer cells. However, the mechanism underlying the effects of morphine on cancer cells remains unclear.

In a study of two different morphine administration regimens in patients with end-stage cancer, longer survival was observed for patients who received intraoperative intrathecal opioids than for those who received intravenous morphine administration, suggesting that a reduction in opioid use may delay tumor progression [5]. Maher retrospectively analyzed 99 patients with non-small cell lung cancer who underwent video-assisted thoracoscopic surgery with lobectomy and found that the recurrence rate of patients within 5 years after lung cancer surgery was positively correlated with the dose of opioid receptor agonists administered during the operation and within 96 h after the operation [6]. In vitro, morphine inhibits the activity and growth of HSC-3 oral cancer cells by inhibiting vascular endothelial growth factor [7] and reduces the growth of MCF-7 breast cancer cells by blocking the cell cycle [8]. However, morphine has been reported to promote tumor cell proliferation by upregulating miR-543 expression in vitro [9]. Therefore, morphine exerts a polarizing effect on tumor cells, but the underlying mechanism remains unclear [10, 11].

Opioid receptors are divided into three subtypes, μ-opioid receptor (MOR), κ-opioid receptor (KOR), and δ-opioid receptor (DOR), among which MOR is the main receptor for commonly used drugs, such as morphine and fentanyl [12]. Further research on the function of MOR showed upregulated MOR expression in the tumor tissues from patients with prostate cancer and that high MOR expression was positively correlated with the metastasis of prostate cancer [13]. Similar results were obtained from studies of esophageal and laryngeal squamous cell carcinoma [14, 15]. Singleton et al. analyzed the clinical specimens from 34 patients with lung cancer and found twofold higher MOR expression in lung cancer tissue than that in adjacent normal lung tissue [16]. The MOR-specific antagonist methylnaltrexone (MNTX) reduces the viability of lung cancer cells by inhibiting Src activation [17].

Mammalian target of rapamycin, mTOR, is typically upregulated in tumor cells to induce proliferation [18]. Phosphoinositide 3 kinase (PI3K)/protein kinase B (AKT), components upstream of mTOR, play important regulatory roles in mTOR activation, and the PI3K/AKT/mTOR signaling pathway is an important downstream target of the Rous sarcoma oncogene cellular homolog (Src) signal transduction pathway [19]. Activation of upstream PI3K/AKT signaling further activates mTOR to promote the growth and division of tumor cells by inducing microtubule growth and other activities [20, 21]. Protein phosphatase 1 (PP1), a specific and effective Src inhibitor, has been shown to bind protein 4.1 N and positively regulates its activity. This complex was reported to inactivate the c-Jun N-terminal kinase (JNK) pathway, a signaling cascade with important roles in cancer pathogenesis, thus inhibiting lung cancer progression [22]. PP1 inhibits C–X–C chemokine receptor 7 (CXCR7)-induced Src phosphorylation and the AKT/mTOR pathway, which inhibits the proliferation of different melanoma cell lines [23, 24]. Deguelin, a naturally occurring rotenoid of the flavonoid family, is an AKT inhibitor with chemopreventive activities that exerts antitumor effects on several cancers. Deguelin impedes carcinogenesis by (1) inducing cell apoptosis, (2) inhibiting tumor cell propagation, and (3) preventing the malignant transformation of tumors through the PI3K/AKT and NF-κB signaling pathways in human lung cancer cells [25–27]. Thus, our current study is the first to determine whether morphine inhibits the growth of H460 cells and regulates apoptosis and the cell cycle in vitro and in vivo. In addition, additional experiments were conducted to investigate whether morphine induces changes in malignant biological behavior through the MOR/Src pathway and to clarify the role of the PI3K/AKT/mTOR signaling pathway in the regulation of H460 cells by morphine.

## Materials and methods

### Materials

Morphine was purchased from Northeast Pharmaceutical Group Shenyang First Pharmaceutical Co., Ltd. (24265669–4, Shenyang, China). The following kits, reagents and proteins were used: a BCA protein assay kit (Sigma–Aldrich); RPMI-1640 (HyClone, USA); DMEM (HyClone, USA); fetal bovine serum (Gibco, USA); trypsin (Gibco, USA); Cell Counting Kit-8 (CCK-8) (TargetMol, USA); methylnaltrexone bromide (TargetMol, USA); the Src inhibitor PP1 (TargetMol, USA); the PI3K/AKT inhibitor deguelin (TargetMol, USA); anti-phospho-Src (Y419; BM4307, BOESTER, China); anti-Src (BM4873, BOESTER, China); anti-phospho-PI3K p85 (Tyr607; YP0765, ImmunoWay, China); anti-PI3K p85 (60225–1-Ig, Proteintech, China); anti-phospho-AKT (Ser473; 66444–1-AP, Proteintech, China); anti-AKT (10176–2-AP, Proteintech, China); anti-phospho-mTOR (Ser2448; 67778–1-Ig, Proteintech, China), anti-mTOR (66888–1-Ig, Proteintech, China), anti-MOR (ab10275, Abcam, UK); anti-GAPDH (ab9385, Abcam, UK); Matrigel (Corning, USA), anti-BCL2 (60178–1-Ig, Proteintech, China); anti-BAX (60267–1-Ig, Proteintech, China); anti-cleaved Caspase-9 (ab2324, Abcam, UK); anti-cleaved Caspase-3 (ab2302, Abcam, UK); and anti-cleaved polyADP-ribose polymerase (PARP) (66520–1-Ig, Proteintech, China). Balb/c female nude mice aged 4–6 weeks were purchased from the Experimental Animal Center of Anhui Provincial Hospital.

### Cell culture

H460 human non-small cell lung cancer cells and BEAS-2B human normal lung epithelial cells were purchased from the National Collection of Authenticated Cell Culture. H460 cells were cultured in RPMI-1640 containing 10% fetal bovine serum, 100 U/ml penicillin, and 100 mg/ml streptomycin at 37 °C in an environment containing 5% CO_2_. BEAS-2B cells were cultured in DMEM containing 10% fetal bovine serum, 100 U/ml penicillin, and 100 mg/ml streptomycin at 37 °C in an environment containing 5% CO_2_. Morphine was dissolved in normal saline (NS) and diluted to several concentrations. MNTX, PP1, and deguelin were dissolved in dimethyl sulfoxide (DMSO) and diluted to several concentrations. Additionally, cells were pretreated with 0.1 μg/μl morphine, 0.1 μg/μl MNTX, 5 μM PP1, or 1 μM deguelin to investigate the mechanisms underlying morphine-induced malignant biological behavior. Cells in the normal control group were cultured with RPMI-1640 medium, NS, or DMSO.

### Cell viability assay

H460 cells were inoculated in 96-well plates at a density of 2 × 10^3^ cells/well. Six replicate wells were prepared for each group. Cells were cultured at 37 °C for 48 h. The supernatant was removed, and 10 μl of CCK-8 reagent were added. Then, the cells were incubated in the dark for 1 h, a standard instrument (Thermo Fisher, Finland) was used to detect the OD at a wavelength of 450 nm, and cell viability was calculated. Each experiment was repeated at least three times.

### Tumor xenograft experiment

Four- to six-week-old female Balb/c nude mice were randomly assigned to control and treatment groups. All mice received a subcutaneous injection of 5 × 10^6^ cells (100 µl of cell suspension) in the back. The treatment groups were subcutaneously injected with morphine (1.5 mg/kg) every other day for 3 weeks based on the results of our preliminary experiment, while the control group received an intraperitoneal injection of NS in the same manner. Tumor growth was observed weekly for 4 weeks. Tumor size was measured using a ruler after treatment. On Day 28, the nude mice were sacrificed, and tumor tissues were isolated and weighed. The tumor volume was calculated using the following formula: volume (mm^3^) = 1/2 × (length × width × width).

### Flow cytometry

Flow cytometry assays were conducted as previously described [7]. Cells were plated into 6-well plates at a density of 3 × 10^5^ cells/well and then incubated with different concentrations of morphine, MNTX, deguelin, or PP1 for 48 h. The treated cells were collected and washed with PBS, resuspended in 500 µl of 1 × binding buffer, and stained with 5 µl of annexin V-FITC (AV) and 7 µl of propidium iodide (PI) for 15 min at 4 °C in the dark. Then, the samples were analyzed using flow cytometry (Agilent NovoCyte, Santa Clara, CA, USA). For the cell cycle analysis, cells were harvested, washed with PBS two times, and then fixed with 70% ethanol overnight at 4 °C. Cells were counterstained with 50 μg/mL PI and 0.1% ribonuclease A (RNase A) in 400 μL of PBS at room temperature in the dark for 30 min. Stained cells were assayed and quantified using flow cytometry (Agilent NovoCyte, Santa Clara, CA, USA). The proliferation index (PI) = (S + G2/M)/(G0/1 + S + G2/M) × 100%. Each experiment was repeated at least three times.

### Wound-healing assay

Wound-healing assays were conducted as previously described [11]. Cell migration was assessed using a wound-healing assay. Cells were cultured in 6-well plates at a density of 5 × 10^5^ cells/well and allowed to grow until reaching 95% confluence. A “wound” was created by scratching the cell monolayer with a 200-µL pipette tip. Then, the cells were cultured with morphine in serum-free medium for 24 h. Images of the wounds were captured at 0 and 24 h (Olympus, Tokyo, Japan). The migration distance was analyzed with ImageJ software. Each experiment was repeated at least three times.

### Transwell assay

Transwell assays were conducted as previously described [22]. The effect of morphine on the migration and invasion of H460 cells was evaluated using Transwell chambers. The chambers were placed in a 24-well culture dish, and 5 × 10^4^ H460 cells resuspended in 100 μL of serum-free medium were then seeded into the upper compartments containing either uncoated or Matrigel-coated membranes. After an incubation for 48 h, the compartments were removed for cleaning, and cells on the lower surface were fixed with methanol and stained with crystal violet. Migration and invasion were assessed by counting the migrated cells on the lower surface under a microscope (Olympus, Tokyo, Japan). The cells were imaged at 100 × magnification and 5 randomly recorded visual fields were captured at 400 × magnification. Each experiment was repeated at least three times.

### Western blot assay

Western blot assays were conducted as previously described [17]. After H460 cells were treated with morphine for 48 h, the collected samples were washed with cold PBS. Then, the cells were lysed in the lysis buffer. After an incubation for 30 min at 4 °C, the samples were centrifuged at 15,000 rpm for 15 min at 4 °C, and the supernatants were collected to determine the protein concentration using a BCA protein assay kit (Sigma–Aldrich). The supernatants of cell homogenates (40 μg of protein equivalent in each sample) were boiled at 100 °C in sample buffer (P0015L, Beyotime, China) for 5 min. The cell extracts were separated on 10% SDS–PAGE gels and transferred to PVDF membranes; membranes were blocked with 5% BSA in TBST. Western blot analyses were performed using primary antibody solutions according to the manufacturer’s instructions. Then, the cells were washed three times with TBST and incubated with secondary antibodies for 1 h at room temperature. The protein bands were detected using the ECL chemiluminescence system. Each experiment was repeated at least three times.

### Immunofluorescence staining

Immunofluorescence assays were conducted as previously described [22]. After growing on coverslips, the cells in 24-well plates cultured at a density of 5 × 10^4^ cells/well were fixed with 4% paraformaldehyde for 20 min, and then, the cells were permeabilized with 0.5% Triton X-100 (Solarbio, Beijing, China) for 20 min at room temperature. Cells were blocked with normal goat serum (Solarbio, China) for 30 min at 37 °C and then incubated overnight at 4 °C with the primary anti-MOR antibody (1:100, Abcam, USA). Then, cells were incubated for 1 h at 37 °C with Cy3-conjugated goat anti-rabbit IgG (Beyotime, China) as the secondary antibody. The nuclei were stained with DAPI (Sangon Biotech, China) for 5 min after washes with PBS. Images were captured using a confocal microscope (Olympus, Tokyo, Japan). Each experiment was repeated at least three times.

### Statistical analysis

The data are presented as the means ± the standard errors of the means. The statistical analyses were performed using paired or unpaired Student’s t-tests, followed by Tukey’s multiple comparison post hoc test, as appropriate. Some data with a normal distribution were analyzed using one-way ANOVA and are presented as the means ± standard deviations. P < 0.05 was considered to indicate a statistically significant difference. GraphPad Prism software (version 8, GraphPad Software, San Diego, CA, USA) was used to calculate statistics and plot the results.

## Results

### Morphine promotes the proliferation, migration and invasion of H460 cells

**Fig. 1:**
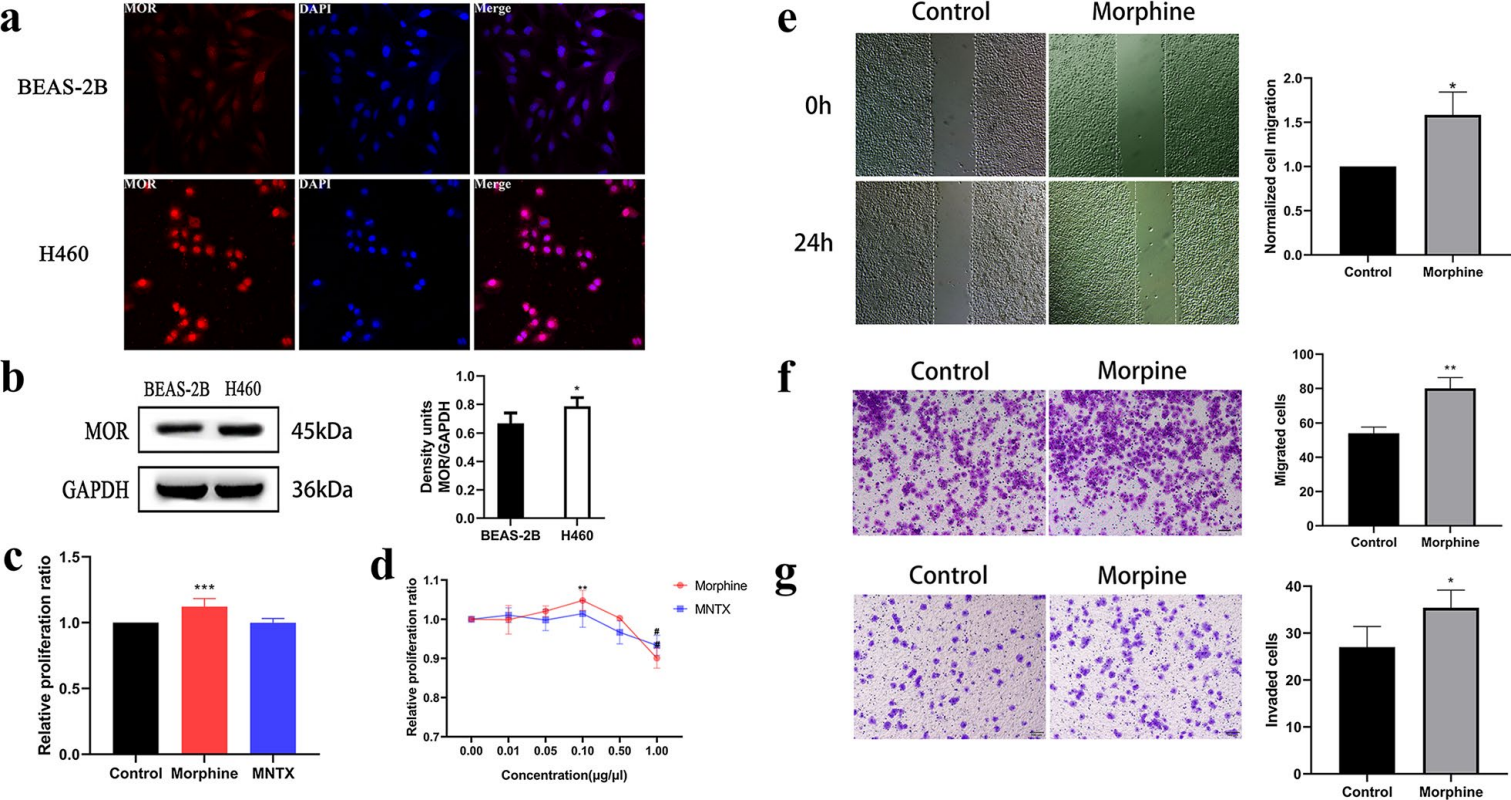
Effects of morphine on the proliferation, migration, and invasion of H460 cells. **a** Representative images of MOR and nuclear immunoreactivity in normal lung epithelial cells, BEAS-2B cells, and H460 cells. **b** Left panel: Expression of the MOR protein in BEAS-2B cells and H460 cells. Right panel: Quantitative analysis of MOR expression shown in the left panel. **c** Quantitative analysis of the proliferation rate of H460 cells after continuous treatment with 0.1 μg/μL morphine and MNTX. The control groups were established by administering NS and DMSO at the same volume. **d** Effects of different concentrations of morphine and MNTX on the relative proliferation rate of H460 cells. **e** Left panel: Representative images of the wound-healing assay using H460 cells treated with morphine. Right panel: Quantitative analysis of normalized cell migration shown in the left panel, **f** Left panel: Representative images of the Transwell assay using H460 cells treated with morphine. Right panel: Quantitative analysis of migrated cells shown in the left panel, **g** Left panel: Representative images of the Transwell assay using H460 cells treated with morphine. Right panel: Quantitative analysis of the invaded cells shown in the left panel. In all experiments, cells were treated with the drugs for 48 h. The drug concentration was 0.1 μg/μL, and the control groups were treated with NS at the same volume in the wound healing and Transwell assays. All data are shown as the means ± standard deviation (SD), *p < 0.05, ^**^p < 0.01, ^***^p < 0.001 and ^#^p < 0.05

We investigated whether MOR is expressed in non-small cell lung cancer (NSCLC) by performing immunofluorescence staining to determine the widespread distribution of MOR in BEAS-2B and H460 cells (Fig. 1a). Moreover, Western blot assays revealed significantly increased MOR expression in H460 cells compared with BEAS-2B cells (Fig. 1b). According to a preliminary experiment and previous results[28], we then treated H460 cells with morphine and the MOR antagonist MNTX for 48 h and found that 0.1 μg/μL morphine promoted the proliferation of H460 cells, while the effect of 0.1 μg/μL MNTX on the proliferation of H460 cells was not significant (Fig. 1c and d). When morphine and MNTX were applied at concentrations of 1.0 μg/μL, the cellular activity of the two groups was significantly inhibited (Fig. 1d), consistent with the results of previous research [10]. Subsequently, the effects of 0.1 μg/μL morphine on the migration and invasion of H460 cells were verified by performing wound-healing and Transwell assays. The scratch test showed that morphine significantly increased the migration of H460 cells after 24 h (Fig. 1e). Moreover, the Transwell assay showed that morphine increased the migration and invasion of H460 cells (Fig. 1f and g) after 48 h in vitro.

### Morphine inhibits the population of cells in G2 phase and the apoptosis of H460 cells

**Fig. 2:**
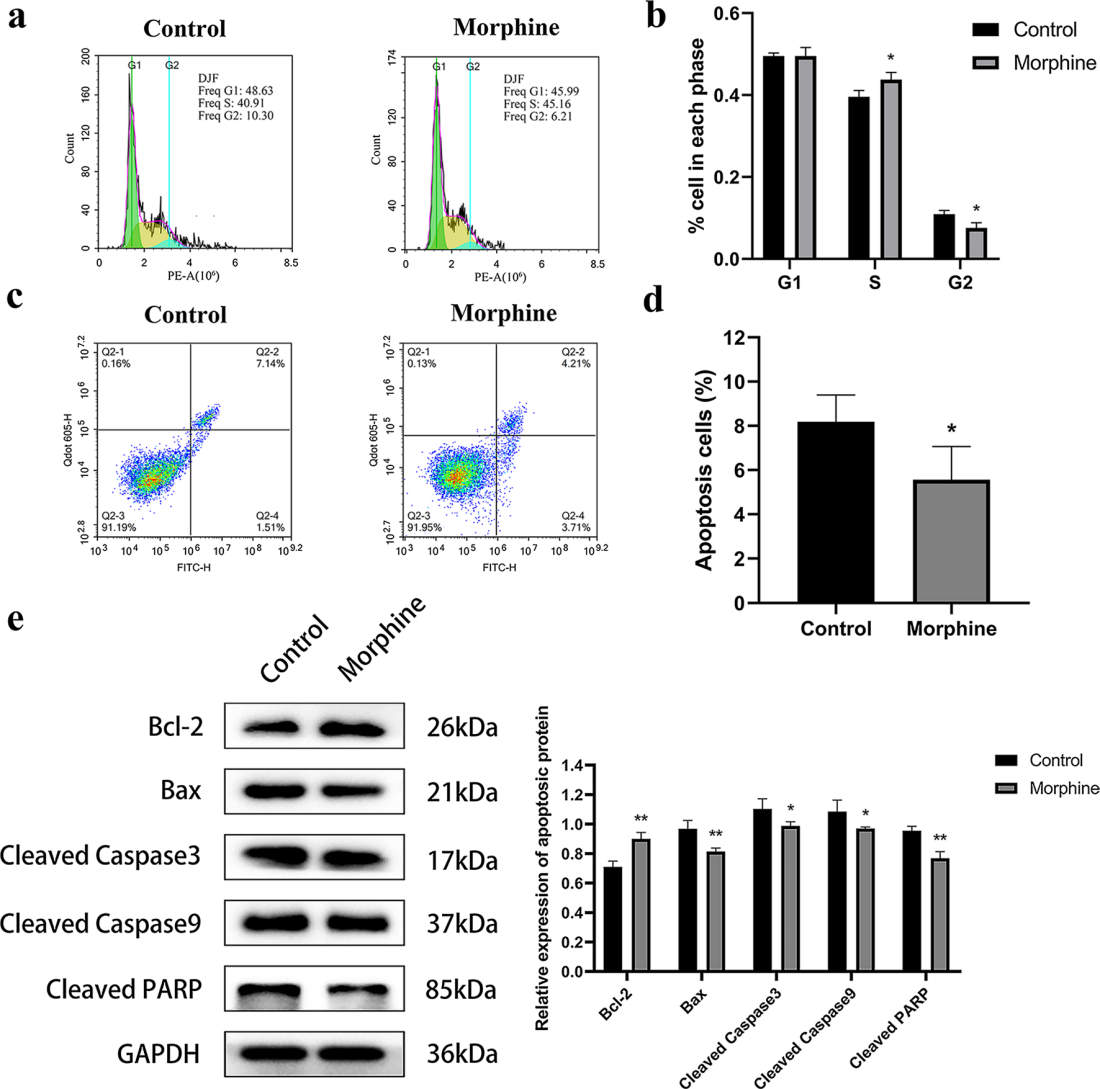
Morphine promotes cell cycle progression through the G_1_/S transition and inhibits apoptosis in H460 cells. **a** Flow cytometry was used to detect the effect of morphine on the cell cycle of H460 cells. **b** Quantitative analysis of the population of cells in each phase shown in (**a)** **c** AV/PI flow cytometry was used to detect the effect of morphine on the apoptosis of H460 cells. **d** Quantitative analysis of the apoptotic cells shown in (**c**). **e** Left panel: Levels of Bcl-2, Bax, cleaved Caspase-3, cleaved Caspase-9, and cleaved PARP were analyzed using Western blotting. Right panel: Quantitative analysis of apoptosis-related protein expression shown in the left panel. H460 cells were treated with 0.1 μg/μL morphine for 48 h, and the control group was treated with NS at the same volume. All data are shown as the means ± SD, *p < 0.05, **p < 0.01

We next measured the effect of morphine on the cell cycle using flow cytometry (Fig. 2a). The population of cells in G2 phase was significantly reduced in the morphine group and a dramatic increase in the number of cells in S phase was observed compared with the control group (Fig. 2b). We next measured the effect of morphine on cell apoptosis using flow cytometry (Fig. 2c), and the population of apoptotic cells in the morphine group was significantly reduced compared with that in the control group (Fig. 2d). In addition, the expression of Bcl-2 protein in the morphine group was increased, while the levels of Bax, cleaved Caspase-3, cleaved Caspase-9, and cleaved PARP were decreased (Fig. 2e). These data collectively suggested that morphine affected cell cycle progression through the S/G2 transition and inhibited the apoptosis of H460 cells.

### Morphine activates the Src/PI3K/AKT/mTOR signaling pathway

**Fig. 3:**
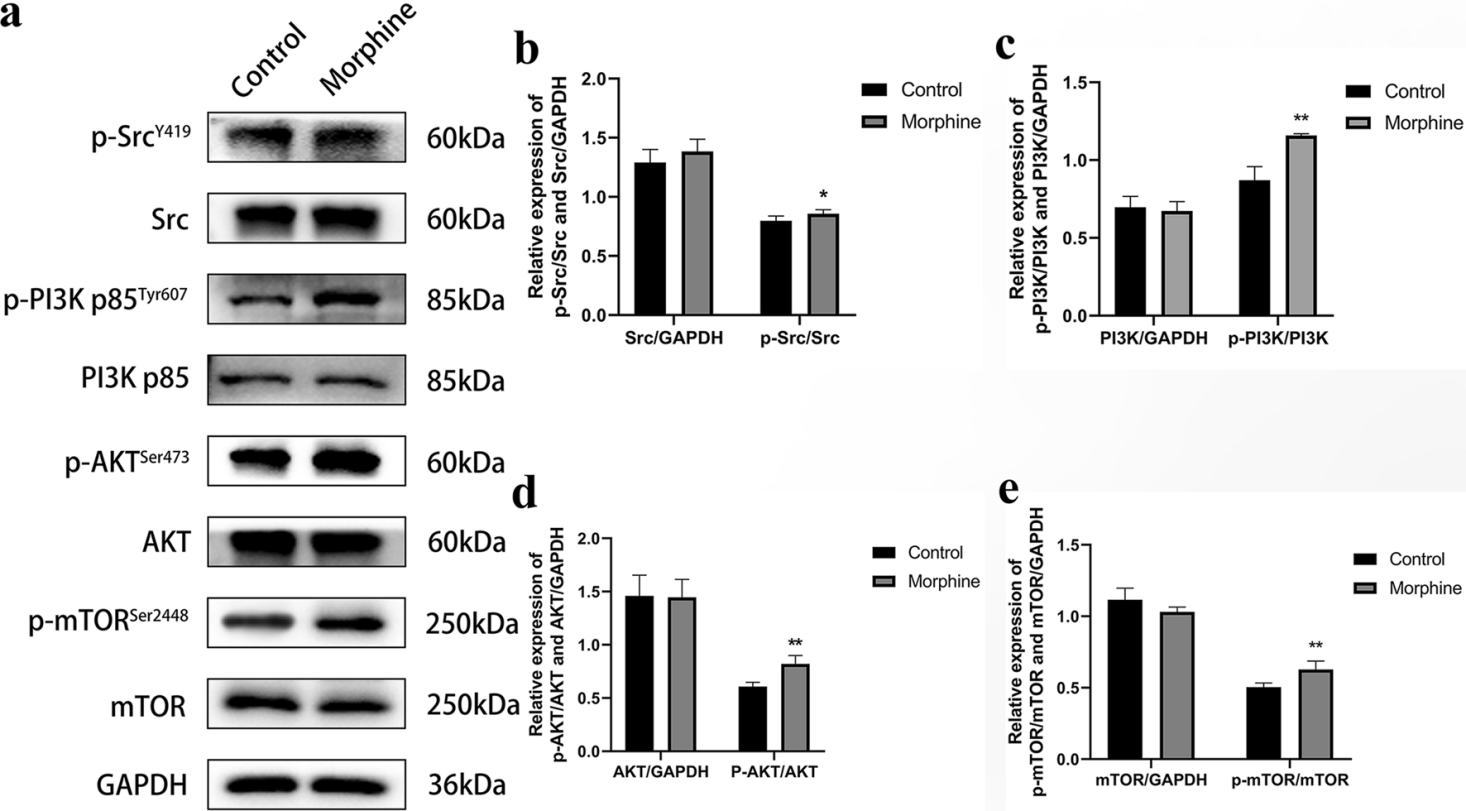
Morphine activates Src kinase and PI3K/AKT/mTOR signaling in vitro. **a** Levels of the total and phosphorylated Src, PI3K, AKT, and mTOR proteins in H460 non-small cell lung cancer cells treated with 0.1 μg/μL morphine for 48 h. **b** Quantitative analysis of Src and phospho-Src levels. **c** Quantitative analysis of PI3K and phospho-PI3K levels. **d** Quantitative analysis of AKT and phospho-AKT levels. **e** Quantitative analysis of mTOR and phospho-mTOR levels. All data are presented as the means ± SD, *p < 0.05, **p < 0.01

The PI3K/AKT/mTOR signaling pathway is involved in the mechanism regulating cell proliferation. We indicated the potential relationship between the morphine-induced increase in malignant biological behavior and the Src/PI3K/AKT/mTOR pathway through Western blot assays (Fig. 3a). Compared with the levels in the control groups, the levels of Src and AKT phosphorylation were increased in the morphine group (Fig. 3b and d), along with the activation of PI3K and mTOR (Fig. 3c and e). This result proved that morphine activated the Src/PI3K/AKT/mTOR signaling pathway, consistent with the results of previous investigations of malignant cell behavior. Thus, morphine may participate in the induction of cell proliferation, migration, and invasion and inhibition of cell apoptosis through the Src/PI3K/AKT/mTOR signaling pathway.

### MOR and Src inhibitors antagonize the morphine-induced proliferation, migration, and invasion of H460 cells

**Fig. 4:**
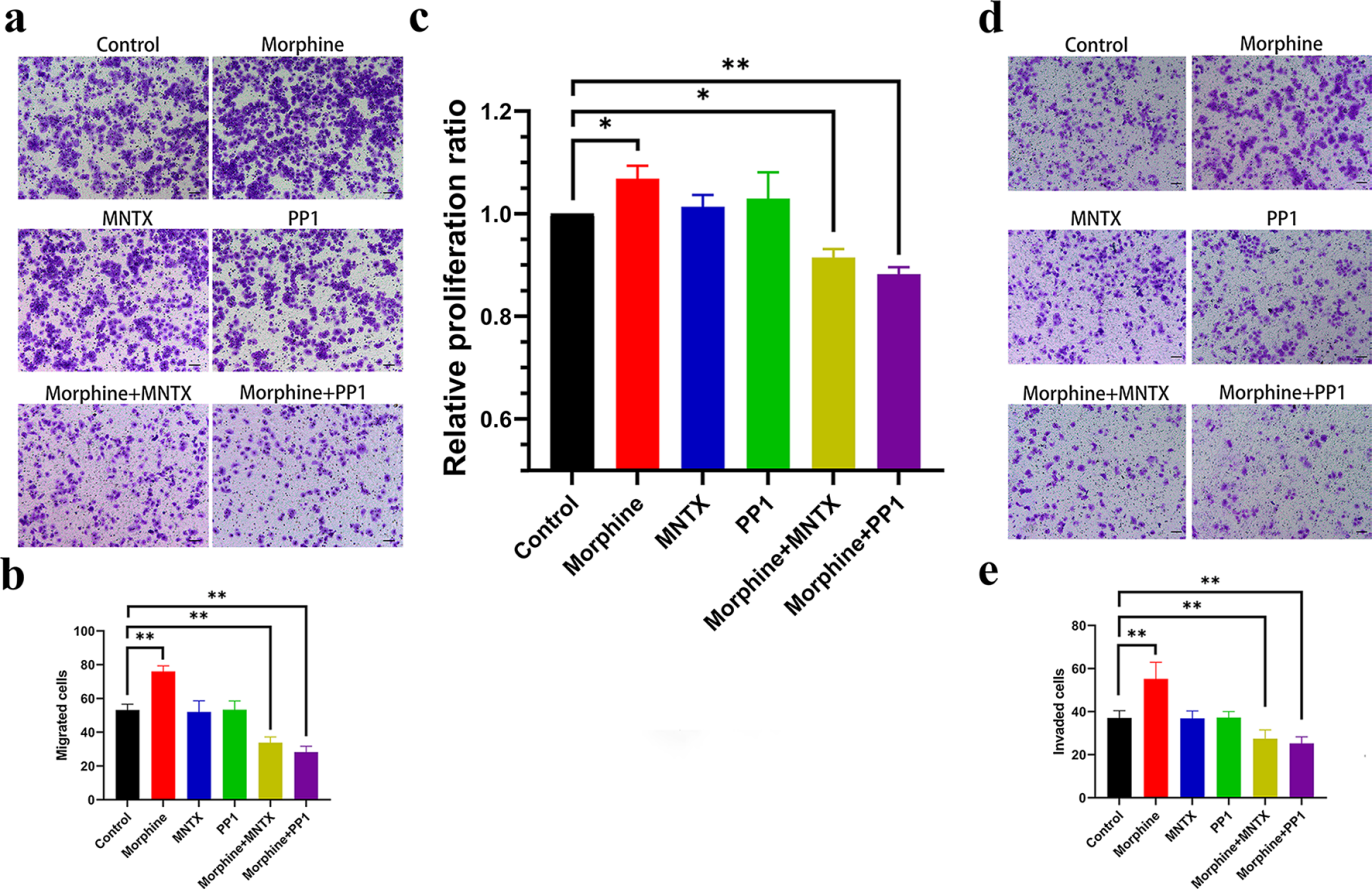
Effects of inhibitors on the proliferation, migration, and invasion of H460 cells. **a** Representative images from the Transwell assay using H460 cells treated with morphine and inhibitors. **b** Quantitative analysis of migrated cells. **c** Quantitative analysis of the proliferation rate of H460 cells after continuous treatment with morphine and inhibitors. **d** Representative images of the Transwell assay with H460 cells that were treated with morphine and inhibitors. **e** Quantitative analysis of invaded cells shown in the preceding panel. In all experiments, cells were treated with the drugs for 48 h. The concentrations of morphine and MNTX were 0.1 μg/μL, the concentration of PP1 was 5 μM, and the control groups were treated with NS and DMSO at the same volumes. All data are presented as the means ± SD, *p < 0.05, **p < 0.01

We treated H460 cells with the MOR antagonist MNTX and the Src inhibitor PP1 to investigate the effect of MOR and Src on the malignant behavior of H460 cells, and the concentration of PP1 was determined using the method described in a previous study [18]. Compared with the control, MNTX significantly inhibited the morphine-induced proliferation of H460 cells (Fig. 4c), and PP1 also exerted obvious inhibitory effects on the proliferation of H460 cells (Fig. 4c). We further explored the effects of MNTX and Src on morphine-induced migration and invasion by performing Transwell migration and invasion experiments (Fig. 4a and d). A quantitative analysis showed that MNTX and PP1 inhibited the migration and invasion of H460 cells treated with morphine compared with the control (Fig. 4b and e). Overall, these data indicated that morphine promoted the proliferation, migration, and invasion of H460 cells through the MOR and Src pathways.

### MNTX and PP1 inhibit the morphine-induced activation of the Src/PI3K/AKT/mTOR signaling pathway

**Fig. 5:**
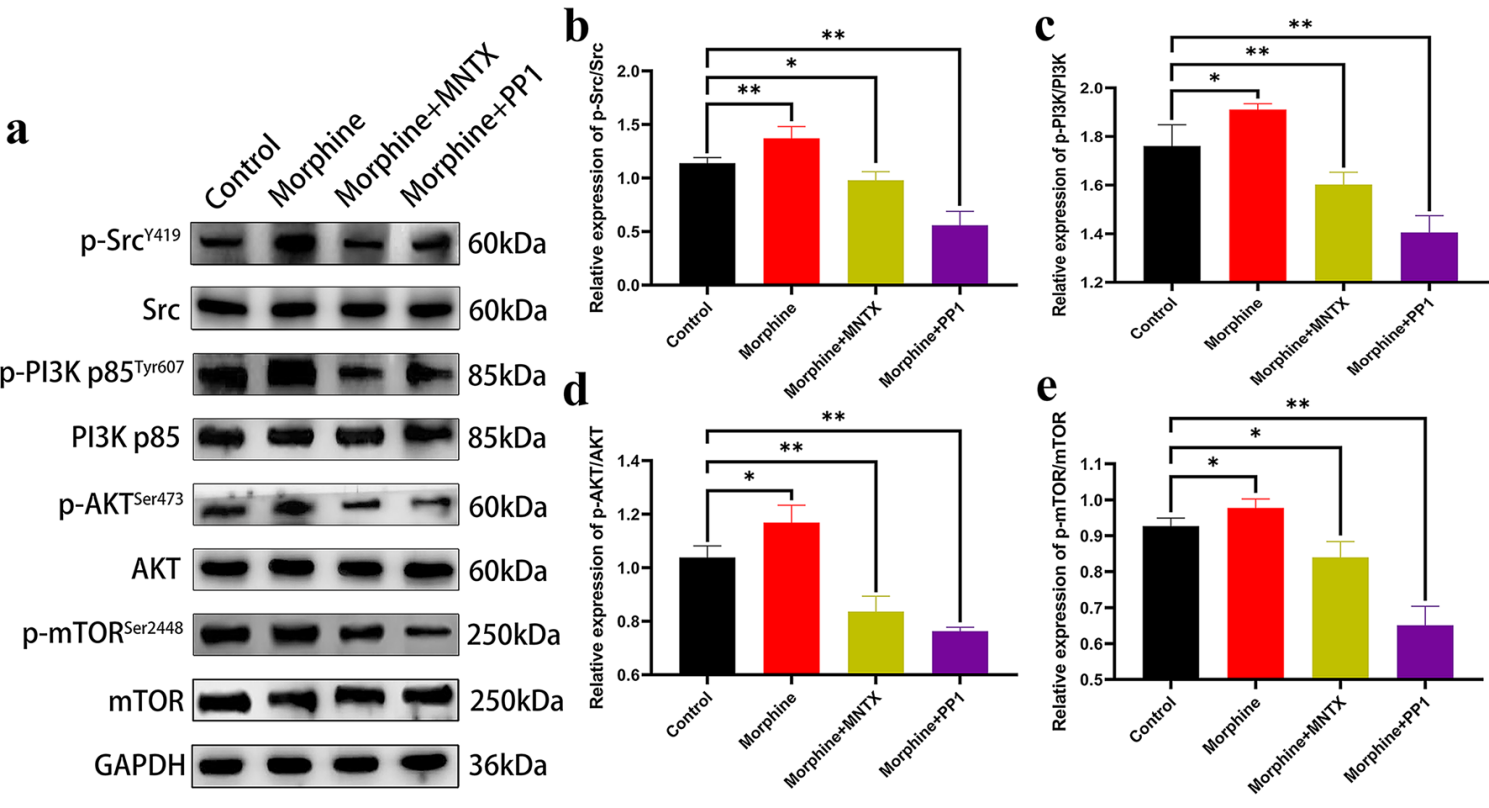
MNTX and PP1 inhibit the morphine-induced activation of the Src/PI3K/AKT/mTOR signaling pathway. **a** Levels of total and phosphorylated Src, PI3K, AKT, and mTOR proteins in H460 non-small cell lung cancer cells treated with drugs. **b** Quantitative analysis of phospho-Src levels normalized to Src levels. **c** Quantitative analysis of phospho-PI3K levels normalized to PI3K levels. **d** Quantitative analysis of phospho-AKT levels normalized to AKT levels. **e** Quantitative analysis of phospho-mTOR levels normalized to mTOR levels, as shown in (**a**). In all experiments, cells were treated with the drugs for 48 h. The concentrations of morphine and MNTX were 0.1 μg/μL, the concentration of PP1 was 5 μM, and the control groups were treated with NS and DMSO at the same volumes. All data are presented as the means ± SD, *p < 0.05, **p < 0.01

First, we performed Western blot analyses to explore the functions of MNTX and PP1 in the Src/PI3K/AKT/mTOR signaling pathway (Fig. 5a). Compared with the control group, MNTX plus morphine inhibited the activation of Src and mTOR (Fig. 5b and e) and more obviously inhibited the activation of PI3K and AKT (Fig. 5c and d). The inhibitory effect of PP1 plus morphine on the activation of the Src/PI3K/AKT/mTOR signaling pathway was basically the same (Fig. 5b-e). Second, compared with the effect of morphine, MNTX and PP1 significantly inhibited the morphine-induce activation of the Src/PI3K/AKT/mTOR pathway (Fig. 5b–e). Based on this result, pharmacological blockade of MOR and Src inhibited the effect of morphine on activating the Src/PI3K/AKT/mTOR pathway.

### Blockade of MOR and Src/PI3K/AKT/mTOR pathways inhibit the cell cycle progression and antiapoptotic effects of morphine

**Fig. 6:**
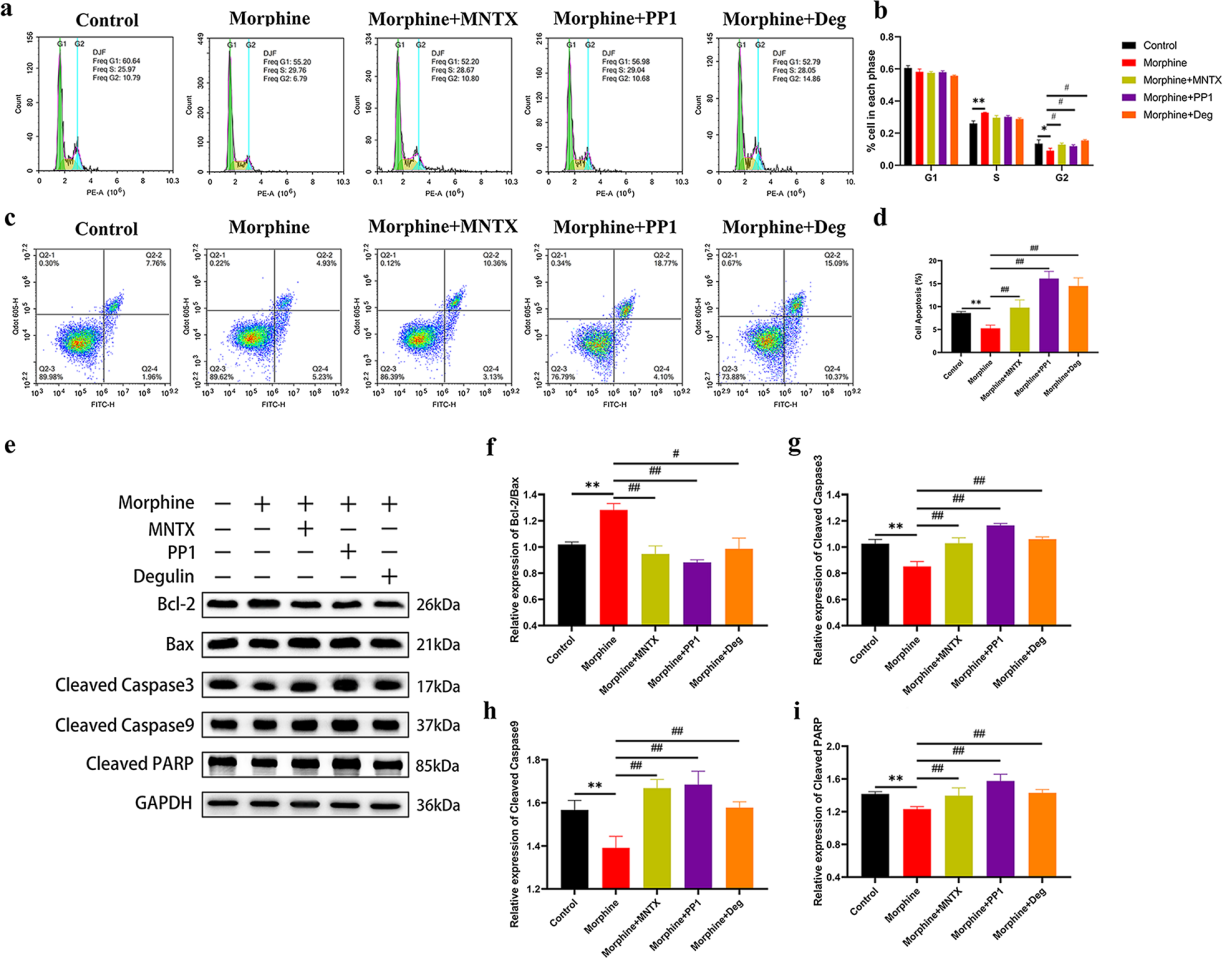
Blockade of the MOR and Src/PI3K/AKT/mTOR pathways inhibit the cell cycle progression and antiapoptotic effects of morphine. **a** Representative images of flow cytometry analyses used to detect the progression of the cell cycle induced by drugs in H460 cells. **b** Quantitative analysis of the cell cycle indicated in (**a**). **c** Representative images from AV/PI flow cytometry used to detect the inhibition of apoptosis induced by drugs in H460 cells. **d** Quantitative analysis of apoptosis indicated in (**c**). **e** The levels of Bcl-2, Bax, cleaved Caspase-3, cleaved Caspase-9, and cleaved PARP were analyzed using Western blotting. **f** Quantitative analysis of Bcl-2/Bax protein expression. **g** Quantitative analysis of the level of the cleaved Caspase-3 protein. **h** Quantitative analysis of the level of the cleaved Caspase-9 protein. **i** Quantitative analysis of the level of the cleaved PARP protein. In all experiments, cells were treated with the drugs for 48 h. The concentrations of morphine and MNTX were 0.1 μg/μL, the concentration of PP1 was 5 μM, and the concentration of deguelin was 1 μM. The control groups were treated with NS and DMSO at the same volumes. All data are shown as the means ± SD, *p < 0.05, **p < 0.01 compared with the control group; ^#^p < 0.05, ^##^p < 0.01 compared with the morphine group

Flow cytometry has previously shown that morphine inhibits apoptosis, while 1 μM deguelin induces apoptosis by blocking PI3K/AKT signaling [19] and results in cell cycle arrest [29]. In our study, flow cytometry showed that groups treated with the inhibitors plus morphine exhibited an inhibition of cell cycle progression compared with the morphine group (Fig. 6a and b). In addition, no significant difference in the proliferation index was observed between the groups that were treated with the inhibitors and the control group. The MNTX plus morphine group exhibited a significant increase in H460 cell apoptosis compared with the morphine treatment group, and the effect was similar to that of deguelin plus morphine (Fig. 6c and d). However, no significant differences were observed between the treatment groups and the control group. The population of apoptotic cells in the PP1 plus morphine group was significantly higher than that in the morphine group (Fig. 6d), indicating that PP1 antagonized the antiapoptotic effect of morphine. Additionally, Western blot analysis showed that H460 cells treated with MNTX, PP1, and deguelin alone and in combination with morphine separately exhibited a decreased Bcl2/Bax protein ratio (Fig. 6f) and increased levels of the cleaved Caspase-3, cleaved Caspase-9, and cleaved PARP proteins (Fig. 6g–i). Therefore, blocking the MOR and Src/PI3K/Akt/mTOR pathways with these drugs inhibited the antiapoptotic effect of morphine, suggesting that morphine modulates apoptosis through the MOR and Src/PI3K/AKT pathways.

### Morphine promotes tumor growth by activating the Src/PI3K/AKT/mTOR pathway in nude mice

**Fig. 7:**
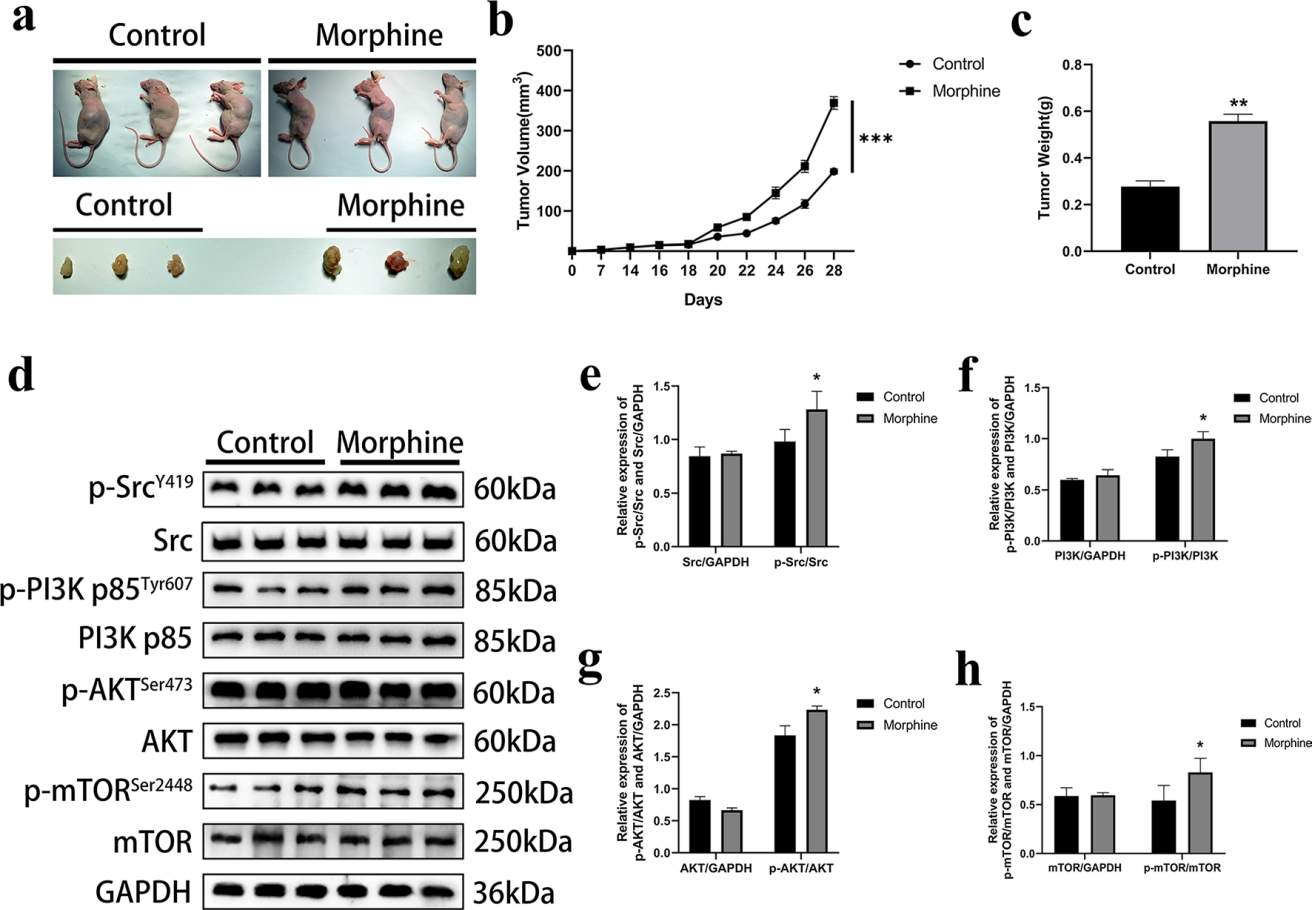
Morphine promotes tumor growth by activating the Src/PI3K/AKT/mTOR pathway in nude mice. **a** The effect of morphine (1.5 mg/kg) on tumorigenesis in nude mice was measured in a xenograft trial. **b** Tumor growth curve in vivo: the horizontal axis represents the growth time and the vertical axis represents tumor size (n = 3; ***p < 0.001). **c** Comparison of tumor tissue weights between the morphine group and the control group (n = 3; **p < 0.01), **d** Levels of total and phosphorylated Src, PI3K, AKT, and mTOR proteins in tumors. **e** Quantitative analysis of Src and phospho-Src levels. **f** Quantitative analysis of PI3K and phospho-PI3K levels. **g.** Quantitative analysis of AKT and phospho-AKT levels. **h** Quantitative analysis of mTOR and phospho-mTOR levels. The control group was treated NS according to body weight. The data are presented as the means ± SD, *p < 0.05

Adult female nude mice aged 4–6 weeks were selected for the tumor formation experiment. The nude mice were divided into 2 groups and subcutaneously injected with morphine and NS on the 7th day. The tumor size in the morphine group was significantly larger than that in the control group on the 28th day (Figs. 7a–c). Western blot analysis confirmed significantly higher activity of the Src/PI3K/AKT/mTOR pathway in the morphine group than that in the control group (Figs. 7d–h). These results clearly reveal that morphine promotes tumor growth and stimulates Src/PI3K/AKT/mTOR signaling in vivo.

## Discussion

In 1986, the World Health Organization proposed a three-step therapy for cancer pain; nonopioid drugs were recommended for mild pain, weak opioid drugs were recommended for moderate pain, and strong opioid drugs were recommended for severe pain. Morphine is still one of the most commonly used opioid drugs [30]. In recent years, morphine has been shown to exert different effects on different tumor cells. J Nguyen showed that morphine did not affect tumorigenesis but promoted the growth of existing tumors and reduced the overall survival rate of mice, and morphine-induced cancer progression may be related to MOR [31]. We found that a low concentration of morphine promotes the malignant biological behavior of H460 non-small cell lung cancer cells in vitro, consistent with previous studies [10, 32]. Other studies have shown that the inhibitory effect of a millimolar concentration of morphine on angiogenesis may be due to its cytotoxicity at this concentration [33, 34]. However, morphine promotes angiogenesis when its concentration in plasma reaches only a level ranging from 2 nM to 3.5 nM [35, 36]. Therefore, we speculate that the inhibitory effect induced by high concentrations of morphine on cells in this experiment was due to its cytotoxicity [37]. As shown in our study, morphine promoted the proliferation of H460 non-small cell lung cancer cells through MOR and significantly increased tumor size in nude mice in vivo, similar to the results obtained by K Gupta et al. [28]. At the same time, we found that morphine can increase the number of cells in S phase and decrease the population of cells in G2 phase, which proves that morphine can block the transition of cells from S to G2 phase, but there is no significant difference in PI, indicating that morphine does not promote cell proliferation through affecting the cell cycle in H460 cells. In addition, as expected, morphine significantly inhibited the apoptosis of H460 cells, increased the Bcl-2/Bax ratio, and significantly decreased Caspase protein expression. Based on these results, morphine inhibits H460 cell apoptosis mainly through the mitochondrial pathway.

Src is an intracytoplasmic tyrosine-specific protein kinase that binds to the cytoplasmic surface of the plasma membrane. When cells are activated by external stimuli (such as growth factors, cytokines, steroid hormones, and drugs that act on G-protein-coupled and adhesion protein receptors), they initiate a signaling cascade that activates Src to produce a wide range of effects [38, 39]. The degree of Src activation is an independent predictor of the prognosis of patients with laryngeal cancer. The greater the degree of Src activation, the higher the level of p-Src (Tyr419), which indicates a worse prognosis for patients with laryngeal carcinoma [40]. Similar conclusions were reported for malignant pleural mesothelioma and hepatocellular carcinoma [41, 42]. PI3K is an intracellular phosphatidylinositol kinase. AKT, also known as protein kinase B, is an important downstream target of the PI3K signal transduction pathway. mTOR is a serine/threonine protein kinase and a downstream effector of PI3K/AKT. P-PI3K p85 (Tyr607), p-AKT (Ser473), and p-mTOR (Ser2448) have been shown to be related to the biological behavior of tumor cells [43–45]. Therefore, the PI3K/AKT/mTOR signaling pathway, one of the most important intracellular signaling pathways, affects the state of downstream effector molecules in many ways. It plays a key role in regulating malignant cell behavior and is closely related to the occurrence and development of a variety of human tumors [46]. In our study, morphine significantly increased the activation of the Src/PI3K/AKT and mTOR proteins, as evidenced by increased phosphorylation of these proteins. In addition, morphine-induced cell proliferation and migration were significantly inhibited after intervention with the MOR antagonist MNTX and the Src inhibitor PP1. MNTX, PP1 and the PI3K/AKT inhibitor deguelin antagonized the antiapoptotic effect of morphine. In contrast to previous studies [17, 18], we found that MNTX and PP1 treatment alone had no effect on the viability of H460 cells, but they exerted an effect in the presence of morphine. We speculated that the continuous effect of morphine is a prerequisite for the effects of MNTX and PP1 and that morphine plays a role in promoting the activation of the MOR and Src/PI3K/AKT/mTOR pathways in H460 cells. Then, we confirmed that morphine promoted the proliferation and migration of H460 cells through the MOR/Src/PI3K/AKT/mTOR pathway.

**Fig. 8:**
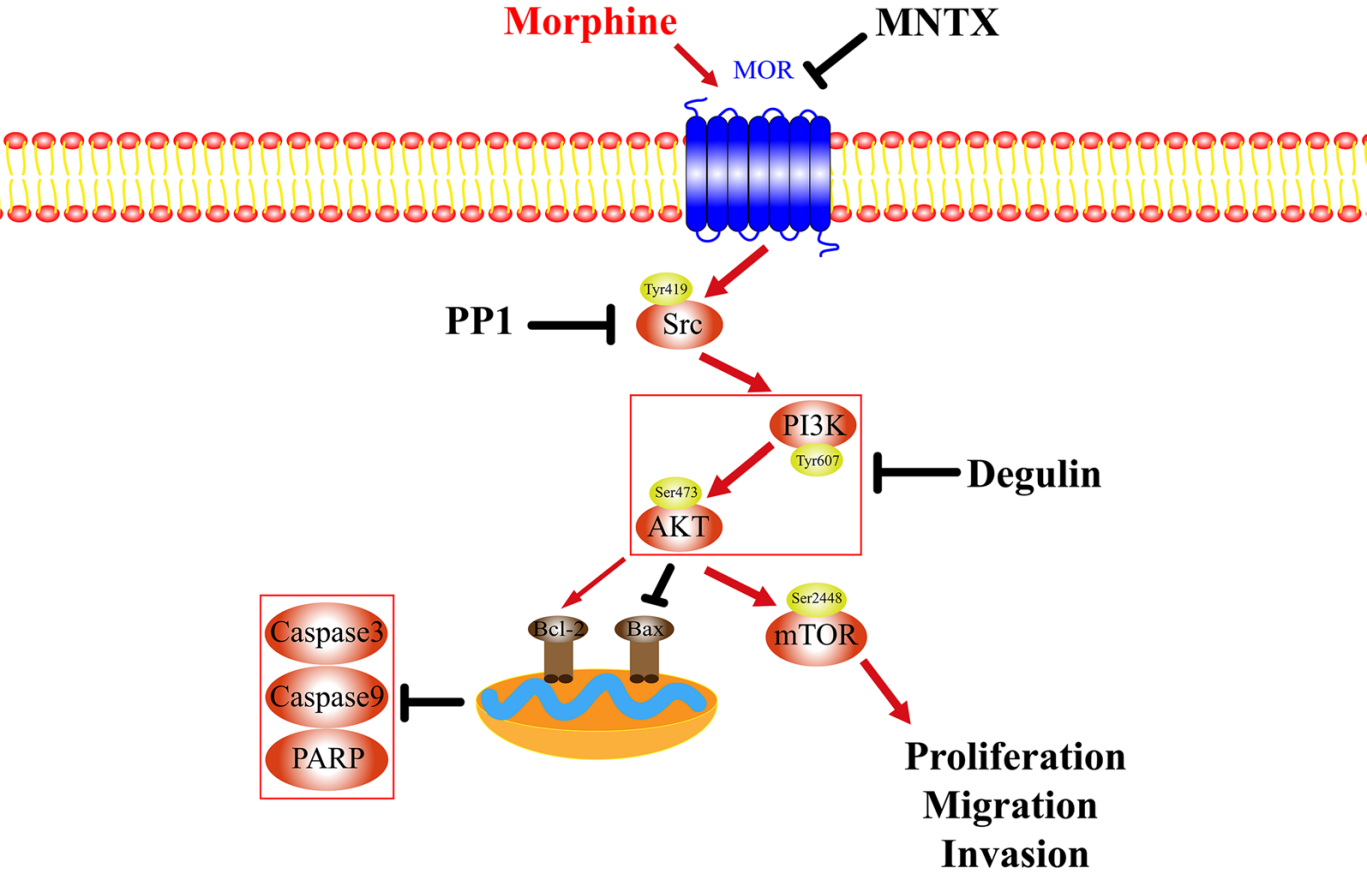
Schematic model illustrating the potential pathway associated with morphine-induced malignant biological behavior in H460 cells. Morphine acts on an MOR located on the H460 cell membrane, leading to Src activation. The activation of Src increases the levels of phospho-PI3K^Tyr607^ and phospho-AKT^Ser473^, which is inhibited by PP1. Src activates the PI3K/AKT pathway, leading to an increase in the expression of proapoptotic factors and a decrease in the expression of antiapoptotic factors. These changes induce Caspase-dependent and mitochondria-mediated apoptotic cell death, which was inhibited by deguelin. Activation of the PI3K/AKT pathway further activates mTOR, leading to an upregulation of factors that induce cell proliferation, migration, and invasion

Therefore, morphine binds specifically to MOR and subsequently activates the Src/PI3K/AKT/mTOR pathway through a cascade effect at low concentrations, thus promoting the proliferation, migration, and invasion of H460 non-small cell lung cancer cells and inhibiting apoptosis in vitro (Fig. 8). Subcutaneous injection of 1.5 mg/kg morphine did not promote tumor formation in nude mice but significantly stimulated tumor growth.

## Conclusions

Morphine promotes the malignant biological behavior of H460 non-small cell lung cancer cells in vivo and in vitro, which provides an experimental basis for the use of morphine in the treatment of pain in patients with advanced lung cancer.

## Supplementary Information

**Additional file 1:** **Figure S1.** Preliminary experiment assessing the effects of morphine and MNTX on the proliferation of H460 cells. We preliminarily verified the effects of previously reported concentrations of morphine and MNTX on the proliferation of H460 cells, and the difference was not statistically significant (Fig. S1a; p>0.05). Then, we increased the drug concentration, and 300 µM morphine promoted the proliferation of H460 cells (Fig. S1b; p<0.01), but MNTX did not induce a significant difference (Fig. S1b; p>0.05). **Figure S2.** The expression of apoptosis-related proteins in tumors from nude mice. In the group subcutaneously injected with morphine, Bcl-2 expression was increased, while the levels of Bax, cleaved Caspase-3, cleaved Caspase-9 and cleaved PARP proteins were decreased. Thus, morphine also inhibits the expression of proapoptotic proteins in vivo. All data are presented as the means ± SEM, *p < 0.05, **p < 0.01 compared with the control group.

## Data Availability

The data used in the current study are available from the corresponding author upon reasonable request.

## Abbreviations

AKT: Protein kinase B
ANOVA: Analysis of variance
AV: Annexin V-FITC
CCK-8: Cell Counting Kit-8
DMSO: Dimethyl sulfoxide
DOR: δ-Opioid receptor
ECL: Enhanced chemiluminescence
KOR: κ-Opioid receptor
MNTX: Methylnaltrexone
MOR: µ-Opioid receptor
mTOR: Mammalian target of rapamycin
NSCLC: Non-small-cell lung cancer
NS: Normal saline
PARP: Poly ADP-ribose polymerase
PI: Propidium iodide
PI3K: Phosphoinositide 3 kinase
PP1: Protein phosphatase 1
PVDF: Polyvinylidene fluoride
RIPA: Radioimmunoprecipitation assay
SDS-PAGE: Sodium dodecyl sulfate–polyacrylamide gel electrophoresis
Src: Rous sarcoma oncogene cellular homolog
TBST: Tris-buffered saline with Tween
