# Supplementary material for: Morphine promotes the malignant biological behavior of non-small cell lung cancer cells through the MOR/Src/mTOR pathway
Source: Cancer Cell Int. 2021 Nov 25;21:622. doi: 10.1186/s12935-021-02334-8 (PMC8613927; doi:10.1186/s12935-021-02334-8)
Supplement: Supplementary file 1 — Additional file 1: Figure S1. Preliminary experiment assessing the effects of morphine and MNTX on the proliferation of H460 cells. We preliminarily verified the effects of previously reported concentrations of morphine and MNTX on the proliferation of H460 cells, and the difference was not statistically significant (Fig. S1a; p>0.05). Then, we increased the drug concentration, and 300 µM morphine promoted the proliferation of H460 cells (Fig. S1b; p<0.01), but MNTX did not induce a significant difference (Fig. S1b; p>0.05). Figure S2. The expression of apoptosis-related proteins in tumors from nude mice. In the group subcutaneously injected with morphine, Bcl-2 expression was increased, while the levels of Bax, cleaved Caspase-3, cleaved Caspase-9 and cleaved PARP proteins were decreased. Thus, morphine also inhibits the expression of proapoptotic proteins in vivo. All data are presented as the means ± SEM, *p < 0.05, **p < 0.01 compared with the control group. [file 12935_2021_2334_MOESM1_ESM.docx]

**
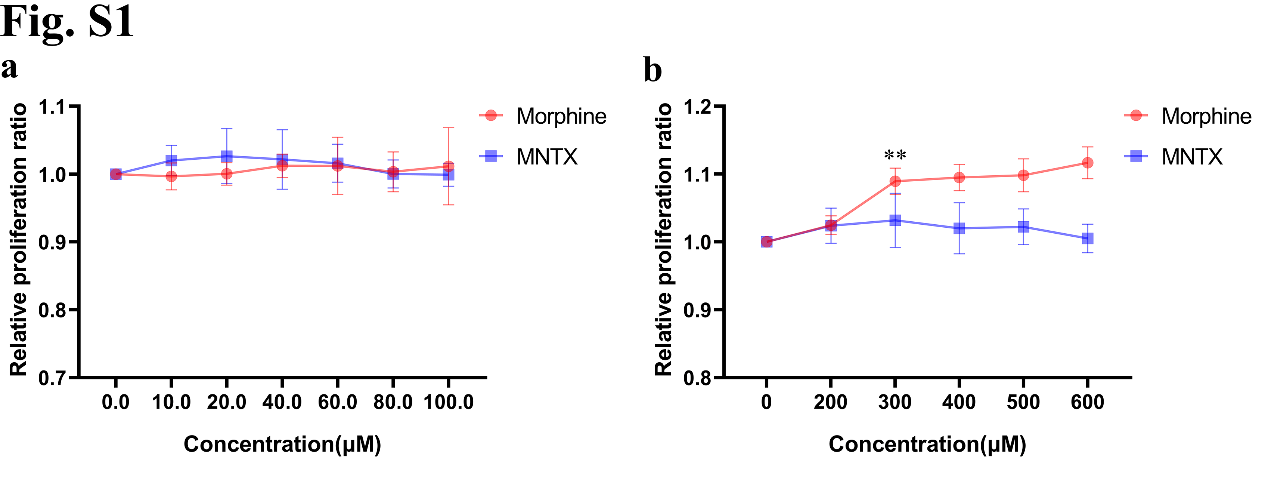
**

**Fig. S1.** Pre-experiment on the effect of morphine and MNTX on the proliferation of H460 cells. We preliminarily verified the effect of reported concentrations of morphine and MNTX on the proliferation of H460 cells, and the results showed that the difference was not statistically significant (Fig. S1.a; p>0.05). Then we increased the drug concentration, and the results showed that 300uM morphine could promote the proliferation of H460 cells(Fig. S1.b; p<0.01), but there was no significant difference in MNTX(Fig. S1.b; p>0.05).


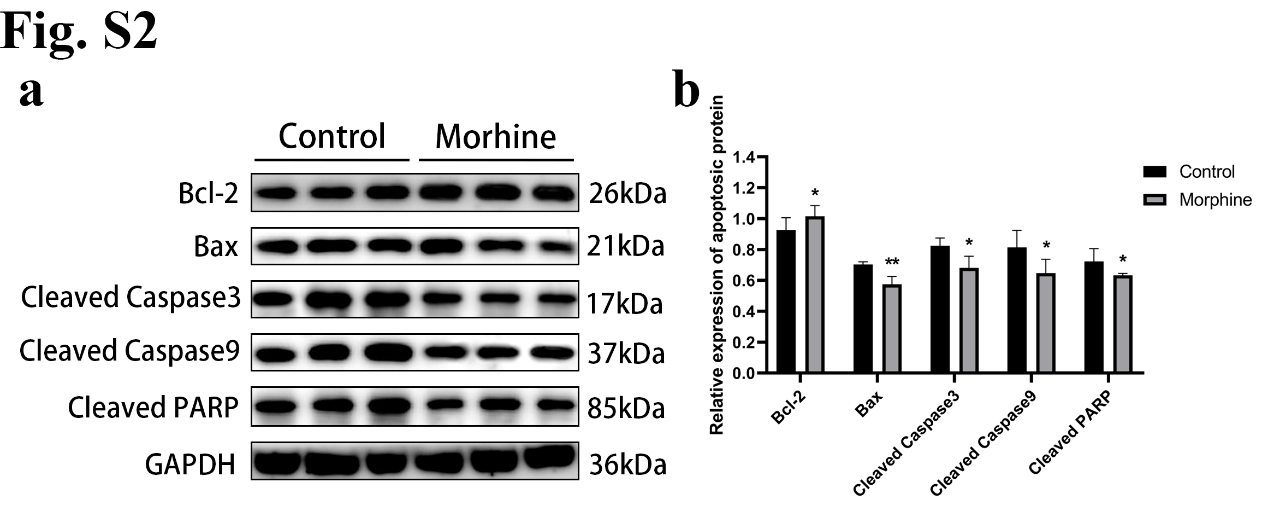


**Fig. S2.** The expression of apoptosis-related proteins in tumors of nude mice. In the subcutaneous injection of morphine group, the expression of Bcl-2 increased, while the expressions of Bax, Cleaved Caspase-3, Cleaved Caspase-9 and Cleaved PARP proteins decreased. This suggests that morphine can also inhibit the proapoptotic protein *in vivo*. All data are shown as the means ± SEM, *p < 0.05, **p < 0.01 versus the control group.
